# Radionuclide Selection Influences Imaging Outcomes in Immuno-PET with a Brain-Penetrating Anti–Amyloid-β Antibody

**DOI:** 10.2967/jnumed.125.271194

**Published:** 2026-03

**Authors:** Sara Lopes van den Broek, Klas Bratteby, Ximena Aguilar, Thuy A. Tran, Stina Syvänen, Dag Sehlin

**Affiliations:** 1 Department of Public Health and Caring Sciences, Uppsala University, Uppsala, Sweden;; 2 Department of Oncology-Pathology, Karolinska Institutet, Stockholm, Sweden; and; 3 Department of Nuclear Medicine and Medical Physics, Theranostics Trial Center, Karolinska University Hospital, Stockholm, Sweden

**Keywords:** Alzheimer disease, immuno-PET, radionuclide, blood–brain barrier, PET

## Abstract

Bispecific antibodies exploiting receptor-mediated transcytosis offer a promising strategy to overcome limited blood–brain barrier permeability in Alzheimer disease immunotherapy and imaging. Lecanemab-Fab8D3 (Lec-Fab8D3), a bispecific amyloid-β (Aβ) antibody with enhanced brain delivery, may complement lecanemab immunotherapy as an immuno-PET imaging agent. Here, we systematically assess how the choice of radionuclide affects PET detection of Lec-Fab8D3 within the brain to evaluate its potential as a companion diagnostic. **Methods:** Lec-Fab8D3 was conjugated to an octadentate derivative of desferrioxamine (DFO*) or NODAGA for ^89^Zr and ^64^Cu radiolabeling, respectively, or directly radioiodinated with ^124^I. PET imaging was performed in the Tg-ArcSwe Aβ mouse model and wild-type (WT) littermates at multiple time points after radiotracer administration, followed by biodistribution, autoradiography, and Aβ quantification to assess brain uptake, specificity, and distribution. **Results:** PET imaging demonstrated high cortical brain uptake of all 3 radiotracers in Tg-ArcSwe mice. Labeling with the metals ^89^Zr and ^64^Cu produced the highest overall brain signal in both Tg-ArcSwe and WT mice. [^89^Zr]Zr-DFO*-Lec-Fab8D3 and [^124^I]I-Lec-Fab8D3 demonstrated the greatest discrimination between Tg-ArcSwe and WT mice, with [^124^I]I-Lec-Fab8D3 exhibiting the most pronounced regional differences. Ex vivo analyses corroborated the PET findings, and immunostaining confirmed radiotracer colocalization with Aβ deposits. **Conclusion:** Immuno-PET imaging with radiolabeled Lec-Fab8D3 enables specific detection of brain Aβ pathology. Because of its residualizing properties, ^89^Zr produced the highest overall signal, whereas ^124^I yielded greater regional contrast, despite lower total brain signal. These findings enhance our understanding of the intrabrain distribution of bispecific antibodies and highlight the importance of radionuclide selection and its impact on immuno-PET outcomes.

## Visual Abstract

**Figure:**
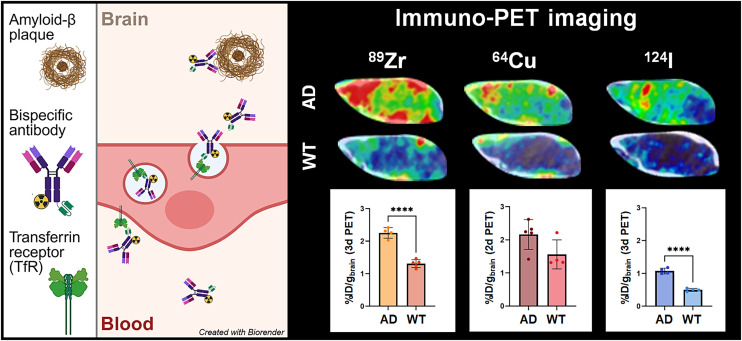

PET has been central to Alzheimer disease (AD) research and drug development. Amyloid PET imaging—using ^11^C-labeled Pittsburgh compound B ([^11^C]PiB) and the ^18^F-labeled florbetaben, flutemetamol, and florbetapir—is a key component in AD clinical trials, as it supports patient selection and the monitoring of therapeutic effects of amyloid-beta (Aβ) antibodies, such as lecanemab, the first approved disease-modifying treatment for AD (1–3). However, amyloid radiotracers do not selectively bind to the Aβ protein but rather to β-sheet–rich fibrillar structures in dense-core amyloid plaques. This results in limited imaging specificity and a mismatch with therapeutic targets, particularly the soluble and diffuse Aβ aggregates recognized by therapeutic antibodies such as lecanemab (4,5).

Immuno-PET, which uses antibodies as PET tracers, addresses this challenge by exploiting the high target specificity of antibodies. However, conventional antibodies have limited ability to cross the blood–brain barrier and therefore require engineering for efficient brain delivery. One approach involves designing bispecific formats that enable receptor-mediated transcytosis across the blood–brain barrier. These bispecific antibodies have dual specificity and bind both to the therapeutic target (Aβ) and to the transferrin receptor (TfR) that facilitates transport into the brain parenchyma (6–9). Bispecific antibodies enable significantly higher brain uptake; and the first anti-Aβ/TfR antibody, trontinemab, is currently under investigation in phase 3 clinical trials for AD. Trontinemab has demonstrated superior brain distribution and reduced side effects compared with conventional antibodies, highlighting the clinical potential of immunotherapies that penetrate the blood–brain barrier (10). In this context, bispecific antibody-based PET tracers could be used as companion diagnostics to improve the specificity of AD imaging and allow evaluation of target engagement and brain distribution of next-generation bispecific immunotherapies.

The most commonly used immuno-PET radionuclides include ^89^Zr (half-life [*t*_1/2_], ∼78 h), ^64^Cu (*t*_1/2_, ∼13 h), and ^124^I (*t*_1/2_, ∼100 h), each offering distinct characteristics that influence their suitability for specific imaging scenarios (Table 1) (9,11,12). In this study, we developed a bispecific variant of lecanemab—lecanemab-Fab8D3 (Lec-Fab8D3)—engineered for TfR-mediated blood–brain barrier transport via the Fab8D3 antibody fragment (13–15). Lec-Fab8D3 was radiolabeled with ^89^Zr, ^64^Cu, or ^124^I to systematically assess how the choice of radionuclide affects PET detection of the antibody within the brain, with the aim of evaluating its potential as a companion diagnostic in AD (Supplemental Scheme 1; supplemental materials are available at http://jnm.snmjournals.org) (16–22).

**TABLE 1.** Properties, Advantages, and Limitations of ^89^Zr, ^64^Cu, and ^124^I

Radionuclide	Half-life	Decay mode*	Positron energy	Advantages	Limitations
^89^Zr	78.4 h (3.3 d)	β^+^ (23%); EC	0.897 MeV	Long half-life matches antibody kinetics; relatively stable chelation	High-energy γ-emission increases radiation dose; requires chelation
^64^Cu	12.7 h	β^+^ (17.9%); β^−^ (39%); EC	0.653 MeV	Short half-life allows earlier imaging; theranostic potential	Moderate half-life may not match antibody kinetics; requires chelation
^124^I	100.2 h (4.2 d)	β^+^ (23%); EC	2.138 MeV	Long half-life matches antibody kinetics; direct labeling	High positron energy reduces resolution; limited availability

* Percentage of decays by each pathway are provided in parentheses.

EC = electron capture.

## MATERIALS AND METHODS

### Mice

The Tg-ArcSwe mouse model, maintained on a C57BL/6 background, harbors the Arctic (E693G) and Swedish (KM670/671NL) amyloid precursor protein (APP) mutations (23). Tg-ArcSwe mice of both sexes (6 males, 7 females) were studied, with WT littermates (4 males, 9 females) as controls. The mice were housed under controlled conditions in an approved facility at Uppsala University, and all described procedures were approved by the Uppsala County Animal Ethics Board (5.8.18–16493/2024), following the regulations of the Swedish Animal Welfare Agency and the European Communities Council Directive of September 22, 2010 (2010/63/EU).

### PET Imaging

The antibody production, modification, and labeling methods used are provided in the supplemental materials. Mice in the [^124^I]Lec-Fab8D3 group were given water supplemented with 0.2% NaI on the day before radiotracer administration to reduce thyroidal uptake of ^124^I. Mice were anesthetized and intravenously injected with 4.92 ± 0.71 MBq of [^89^Zr]Zr-DFO*-Lec-Fab8D3 (Tg-ArcSwe, *n* = 4; WT, *n* = 5), 31.23 ± 5.26 MBq of [^64^Cu]Cu-NODAGA-Lec-Fab8D3 (Tg-ArcSwe, *n* = 5; WT, *n* = 4), or 4.68 ± 0.72 MBq of [^124^I]I-Lec-Fab8D3 (Tg-ArcSwe, *n* = 4; WT, *n* = 4), corresponding to 0.86 ± 0.11, 0.55 ± 0.02, and 1.20 ± 0.07 mg antibody per kilogram of body weight, respectively. Dynamic PET images were acquired for 30 min at 9 and 24 h after tracer injection and for 60 min at 48 or 72 h after injection, using a PET/MRI scanner (nanoScan PET-3T MRI; Mediso Medical Imaging Systems). The bed was then transferred to a 4-head SPECT/CT scanner (nanoScan SPECT/CT; Mediso Medical Imaging Systems) for a 6 min 80-kV CT scan.

PET data were reconstructed into 10-min frames using the ordered-subsets expectation maximization 3-dimensional algorithm, and CT raw files were reconstructed using filter backprojection. All subsequent processing of the PET and CT images was performed using Amide 1.0.6 imaging software (24). The CT scan was manually aligned with an MRI-based mouse brain atlas (25) containing outlined regions of interest (whole brain, hippocampus, striatum, thalamus, cortex, and cerebellum). The PET image was then aligned with the CT and MRI atlas, and PET data were quantified. In all PET experiments, mice were scanned in a randomized order.

### Ex Vivo Biodistribution

After 48 or 72 h, mice were anesthetized with isoflurane, and a terminal blood sample was taken from the heart, followed by transcardial perfusion with 40 mL of 0.9% NaCl for 2.5 min to clear the blood from the organs. Organs (brain, lung, liver, kidney, spleen, heart, femoral muscle, femoral bone, pancreas, skull, and thyroid) were isolated from all scanned mice to evaluate radiotracer biodistribution. The brain was divided into left and right hemispheres, and the left hemisphere was further divided into cerebrum (brain) and cerebellum. All brain samples were immediately frozen on dry ice. The radioactivity in the samples was measured with a γ-counter (Wallac 2480 Wizard; PerkinElmer). Antibody concentrations were calculated by dividing the radioactivity per gram of tissue by the total injected radioactivity and expressed as a percentage of the injected dose per gram of tissue.

### Autoradiography

After PET scanning and perfusion, the spatial brain distribution of the radiotracers was analyzed with autoradiography. Right-hemisphere sagittal cryosections (20 µm) from all scanned mice were exposed together with a radioactive standard to phosphor imaging plates (PerkinElmer). After 3–7 d, the phosphor plates were scanned with an Amersham Typhoon imager (Cytiva) at 50-µm resolution. Images were processed in ImageJ.

### Immunofluorescence

Sagittal brain tissue sections (20 µm) from all PET scanned mice were fixed in ice-cold methanol, rinsed in phosphate-buffered saline (PBS) and blocked with 5% normal goat serum and 0.4% Triton X-100 in PBS, followed by three 5-min washes in PBS at room temperature. To detect the injected radiotracers, the sections were incubated for 1 h at room temperature with Alexa Fluor 647-conjugated AffiniPure F(ab’)2 fragment goat antihuman IgG, F(ab’)2 (fragment specific (109-606-006, NordicBiosite), diluted 1:200 in PBS 0.05% polysorbate 20, and then washed 3 times for 5 min each at room temperature. To label Aβ deposits, sections were incubated for 15 min at room temperature with the luminescent conjugated oligothiophene HS-84 (26) at 50 nM in PBS 0.05% polysorbate 20, followed by three 5-min washes in PBS at room temperature. Sections were mounted with 4’,6-diamidino-2-phenylindole mounting medium and imaged using a Zeiss Observer Z.1 microscope with ZEN 3.7 software (Carl Zeiss Microimaging GmbH).

### Statistical Analysis

Statistical analyses were performed in Prism 10.1.0 (GraphPad Software). The results were reported as mean ± SD, and statistical assessment was conducted using an unpaired *t* test or a 2-way ANOVA with multiple comparisons test.

## RESULTS

### PET Imaging with ^89^Zr-, ^64^Cu-, and ^124^I-Labeled Lec-Fab8D3

**FIGURE 1.**
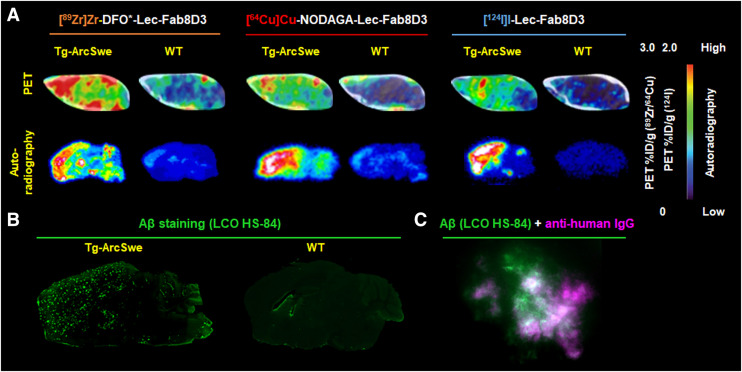
(A) Sagittal PET images of brain 48 or 72 h after injection, show cortical uptake of [^89^Zr]Zr-DFO*-Lec-Fab8D3 (left), [^64^Cu]Cu-NODAGA-Lec-Fab8D3 (middle), and [^124^I]I-Lec-Fab8D3 (right) in Tg-ArcSwe mice. (B and C) PET images are corresponding autoradiography images of sagittal brain sections, confirming radiotracer accumulation in cortical regions associated with Aβ plaque deposition. Brain uptake is presented as percentage of injected dose per gram of brain. (B) Tg-ArcSwe and WT brain sections stained for Aβ pathology with aggregate specific luminescent conjugated oligothiophene (LCO) stain HS-84. (C) Combined Aβ (green; HS-84) and antihuman IgG staining (magenta) show colocalization of injected Lec-Fab8D3 tracer and Aβ plaques.

Lec-Fab8D3 was conjugated with an octadentate derivative of desferrioxamine (DFO*)-NCS or p-NCS-benzyl-NODAGA and radiolabeled with [^89^Zr]Zr-oxalate or [^64^Cu]CuCl_2_, or directly with [^124^I]NaI, producing tracers of high radiochemical purity and yield (Supplemental Table 1). Preserved binding to Aβ and TfR after labeling was confirmed with enzyme-linked immunosorbent assay (Supplemental Fig. 1). PET imaging was performed in Tg-ArcSwe mice (age, 18–20 mo) and age-matched WT controls at 9, 24, and 72 h after administration of [^89^Zr]Zr-DFO*-Lec-Fab8D3 and [^124^I]I-Lec-Fab8D3 or at 9, 24, and 48 h after administration of [^64^Cu]Cu-NODAGA-Lec-Fab8D3 because of the shorter half-life of ^64^Cu. PET images representing radiotracer distribution in the brain at the last time point (48 or 72 h) clearly visualized Aβ-rich brain regions in Tg-ArcSwe mice, regardless of the radionuclide used (Fig. 1A; Supplemental Fig. 2). The highest radioactivity was detected in Tg-ArcSwe mice injected with [^89^Zr]Zr-DFO*-Lec-Fab8D3 or [^64^Cu]Cu-NODAGA-Lec-Fab8D3. In contrast, the lowest radioactivity was found in WT mice injected with [^124^I]I-Lec-Fab8D3.

Ex vivo autoradiography further supported the in vivo PET results (Fig. 1A; Supplemental Figs. 2 and 3). Here, radiotracer accumulation was observed in the cortex, hippocampus and thalamus of Tg-ArcSwe mice, consistent with the pattern of Aβ deposition (Fig. 1B). Interestingly, WT mice injected with radiometal (^64^Cu or ^89^Zr) labeled Lec-Fab8D3 displayed a faint but clear radioactive signal in the frontal cortex and central regions of the brain, despite absence of Aβ deposits in WT mice (Fig. 1A; Supplemental Figs. 2 and 3). Autoradiography revealed lower signal in the cerebellum of the mice injected with [^124^I]I-Lec-Fab8D3, consistent with low cerebellar Aβ deposition in Tg-ArcSwe mice (Fig. 1B, Supplemental Fig. 4) (27). To confirm antibody engagement with Aβ pathology, the administered Lec-Fab8D3 was detected by antihuman IgG immunostaining, which showed a close colocalization with Aβ pathology (Fig. 1C).

PET imaging at earlier time points (i.e., 9 and 24 h), provided a less distinct difference between the Tg-ArcSwe and WT mice, attributable to the high concentration of radiolabeled antibody remaining in the bloodstream, which resulted in an elevated background signal (Supplemental Fig. 2).

### Radiotracer Clearance

**FIGURE 2.**
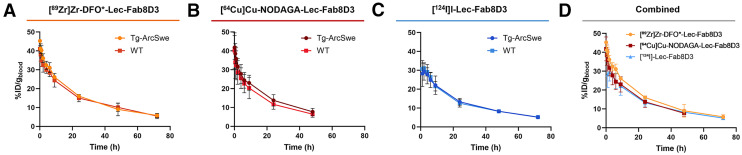
Blood elimination curves of [^89^Zr]Zr-DFO*-Lec-Fab8D3 (0–72 h) (A), [^64^Cu]Cu-NODAGA-Lec-Fab8D3 (0–48 h) (B), [^124^I]I-Lec-Fab8D3 (0–72 h) (C) in Tg-ArcSwe and WT mice and all 3 radiotracers (D) in Tg-ArcSwe mice.

Blood radiotracer concentrations (Figs. 2A–2D) revealed similar clearance for all radiolabeled Lec-Fab8D3 variants, with an average terminal blood concentration of 5.63 ± 0.01, 7.18 ± 0.02, and 4.67 ± 0.02 %ID/g for ^64^Cu-, ^89^Zr-, and ^124^I-labeled antibodies, respectively. Of note, the mice who were injected with [^64^Cu]Cu-NODAGA-Lec-Fab8D3 were sacrificed 1 d earlier.

### Aβ-Related Brain Retention and Brain-to-Blood Ratios

**FIGURE 3.**
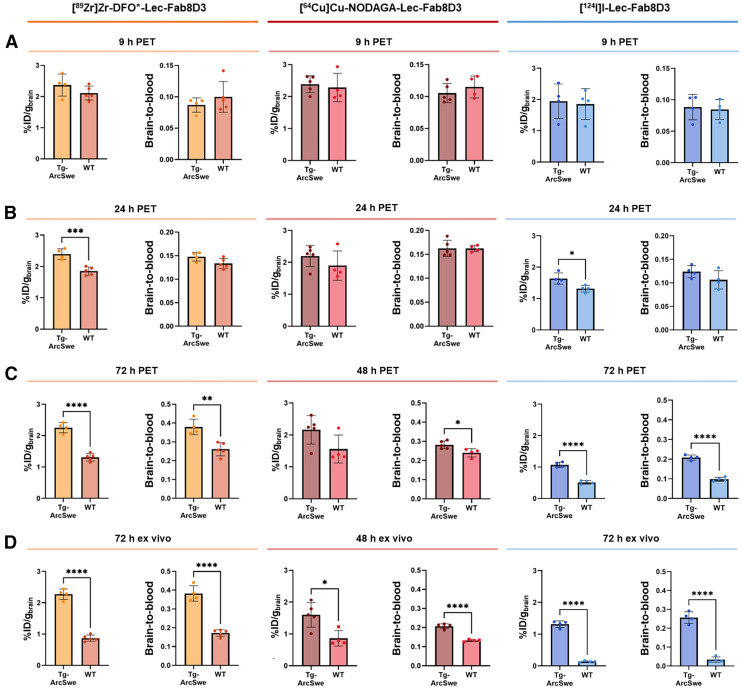
Quantification of brain concentration, expressed as percentage of injected dose per gram of brain and brain-to-blood ratio derived from PET imaging after 9 h (A), 24 h (B), and 48 h or 72 h (C) after radiotracer administration and ex vivo γ-counting in perfused postmortem brain tissue (D). Data represent mean ± SD. *P ≤ 0.05, **P ≤ 0.01, ***P ≤ 0.001, ****P < 0.0001.

PET quantification showed no difference between WT and Tg-ArcSwe mice in brain retention or brain-to-blood ratio at 9 h after radiotracer administration (Fig. 3A). With time, all radiotracers displayed an increased difference between Tg-ArcSwe and WT mice, expressed both as absolute brain concentrations and brain-to-blood ratios (Fig. 3; Supplemental Table 2). Specifically, [^89^Zr]Zr-DFO*-Lec-Fab8D3 and [^124^I]I-Lec-Fab8D3 were found at higher concentrations in the brains of Tg-ArcSwe mice compared with WT mice at 24 h after administration. As unbound tracer washed out from the brain, the difference between Tg-ArcSwe and WT mice increased to about 2-fold at 72 h. A similar trend was noted for [^64^Cu]Cu-NODAGA-Lec-Fab8D3, although statistical significance was not reached because of a high variability between mice (Figs. 3B and 3C). However, at the last PET imaging time point, all 3 radionuclides displayed a higher brain-to-blood ratio in Tg-ArcSwe mice compared with WT mice. Notably, compared with the radioiodinated antibody, the radiometal-labeled antibodies exhibited higher overall brain concentrations in both Tg-ArcSwe and WT mice.

Regional analysis of the PET scans performed 48 or 72 h after antibody administration confirmed higher uptake in Tg-ArcSwe mice compared with WT mice across multiple brain regions for all 3 radiotracers (Supplemental Fig. 5). Regions with high Aβ pathology (e.g., cortex, hippocampus) showed the largest differences, whereas the cerebellum and brainstem, which typically have low Aβ burden, showed small differences.

Ex vivo analysis (i.e., γ-counting of perfused brains collected after the final PET scans) further confirmed the increased brain uptake and brain-to-blood ratios in Tg-ArcSwe mice compared with WT mice for all 3 radionuclides (Fig. 3D). The most distinct differences in absolute brain concentrations were observed for [^89^Zr]Zr-DFO*-Lec-Fab8D3 and [^124^I]I-Lec-Fab8D3, whereas [^64^Cu]Cu-NODAGA-Lec-Fab8D3 showed a less pronounced but still detectable difference between Tg-ArcSwe and WT mice. Consistent with the PET results, the variation between mice injected with [^64^Cu]Cu-NODAGA-Lec-Fab8D3 was reduced after correcting for blood concentration, resulting in a higher brain-to-blood ratio in Tg-ArcSwe mice compared with WT mice.

### Reference Region Analysis

**FIGURE 4.**
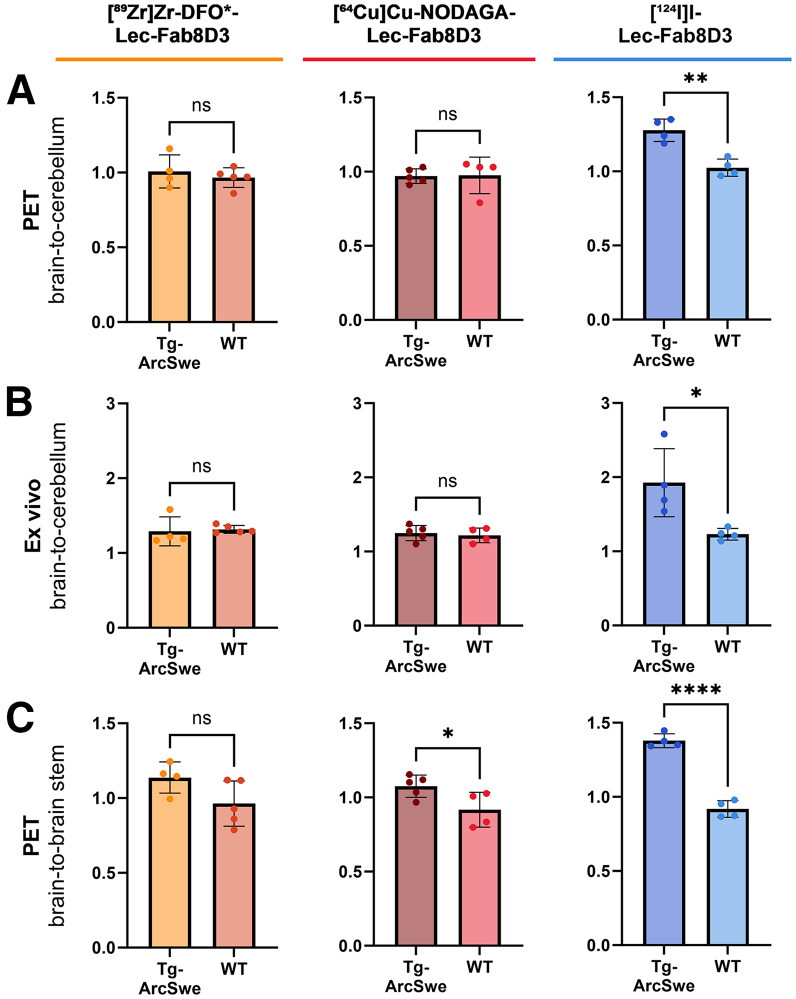
PET-derived (A) and corresponding ex vivo–derived (B) brain-to-cerebellum ratios in Tg-ArcSwe and WT mice 72 h after administration of [^89^Zr]Zr-DFO*-Lec-Fab8D3, 48 h after administration of [^64^Cu]Cu-NODAGA-Lec-Fab8D3, and 72 h after administration of [^124^I]I-Lec-Fab8D3. (C) PET-derived brain-to-brainstem ratios in Tg-ArcSwe and WT mice 72 h after administration of [^89^Zr]Zr-DFO*-Lec-Fab8D3, 48 h after administration of [^64^Cu]Cu-NODAGA-Lec-Fab8D3, and 72 h after administration of [^124^I]I-Lec-Fab8D3. Data represent mean ± SD. ns = not significant. *P ≤ 0.05, **P ≤ 0.01, ****P < 0.0001.

The cerebellum is often used as an amyloid PET reference region because of its lower Aβ accumulation in patients with AD (28). Similarly, Tg-ArcSwe mice used in this study displayed lower Aβ levels in the cerebellum (Supplemental Fig. 4). PET-derived whole brain–to-cerebellum (hereafter referred to as brain-to-cerebellum) ratios calculated from the terminal PET scans were approximately 1 in both Tg-ArcSwe and WT mice for the radiometal-labeled [^89^Zr]Zr-DFO*-Lec-Fab8D3 and [^64^Cu]Cu-NODAGA-Lec-Fab8D3. In contrast, [^124^I]I-Lec-Fab8D3 showed a substantially higher brain-to-cerebellum ratio in Tg-ArcSwe compared with WT mice (Fig. 4A; Supplemental Table 3). Similarly, ex vivo brain-to-cerebellum ratios were slightly above 1 for mice who received [^89^Zr]Zr-DFO*-Lec-Fab8D3 or [^64^Cu]Cu-NODAGA-Lec-Fab8D3, with no genotype-dependent differences. Mice injected with [^124^I]I-Lec-Fab8D3 showed a slightly elevated ex vivo brain-to-cerebellum ratio compared with PET, but with a preserved genotype-dependent difference (Fig. 4B; Supplemental Fig. 3B). A broader region of interest–to-cerebellum analysis revealed no significant differences between Tg-ArcSwe and WT mice scanned with either [^89^Zr]Zr-DFO*-Lec-Fab8D3 or [^64^Cu]Cu-NODAGA-Lec-Fab8D3. In contrast, Tg-ArcSwe mice scanned with [^124^I]I-Lec-Fab8D3 showed higher ratios across all analyzed regions compared with WT mice (Supplemental Figs. 6A–6C).

The cerebellum was chosen as the reference region because of Aβ quantification in the tissue. Another approach to identify a reference region is to use PET-quantified tracer uptake. Regional PET quantification identified the brainstem as exhibiting the lowest overall uptake in Tg-ArcSwe mice as well as the smallest difference between WT and Tg-ArcSwe mice (Supplemental Figs. 6A–6C). Brain-to-brainstem ratios improved genotype discrimination for all radiotracers compared with cerebellum-based ratios. The radiometal-labeled tracers tended to show a higher brain-to-brainstem ratio in Tg-ArcSwe mice compared with WT mice, but a significant difference was observed only for [^64^Cu]Cu-NODAGA-Lec-Fab8D3. Mice injected with [^124^I]I-Lec-Fab8D3 demonstrated the largest difference between the genotypes, with a 50% higher PET-derived brain-to-brainstem ratio in Tg-ArcSwe mice compared with WT mice (Fig. 4C; Supplemental Figs. 6D–6F) and an even greater discrimination with ex vivo autoradiography (Supplemental Fig. 3C). Again, PET-derived region-specific ratios using the brainstem as a reference region were calculated for each radiotracer. [^124^I]I-Lec-Fab8D3 demonstrated genotype differences for all studied regions, whereas radiometal-labeled Lec-Fab8D3 showed smaller differences between Tg-ArcSwe and WT mice that were only significant for the thalamus and caudate putamen (Supplemental Figs. 6D–6F).

### Retention of Radiometal-Labeled Lec-Fab8D3 Variants in Peripheral Organs

**FIGURE 5.**
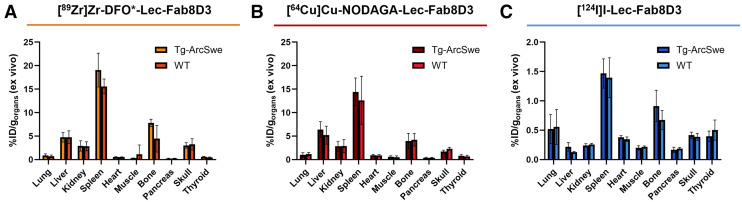
Ex vivo biodistribution to peripheral organs of [^89^Zr]Zr-DFO*-Lec-Fab8D3 (72 h after injection) (A), [^64^Cu]Cu-NODAGA-Lec-Fab8D3 (48 h after injection) (B) and [^124^I]I-Lec-Fab8D3 (72 h after injection) (C). Data represent mean ± SD.

Ex vivo radiotracer biodistribution to peripheral organs revealed higher overall concentrations of the radiometal-labeled antibodies compared with the radioiodinated variant (Figs. 5A–5C). For all 3 radiolabeled variants of Lec-Fab8D3, the highest uptake was observed in the spleen, which is consistent with previous findings and can be attributed to the TfR expression on immature erythroid cells and reticulocytes that are highly abundant in the spleen (15,29–31). Elevated signals were also detected in the bone and skull, likely attributable to the antibody interaction with TfR expressed in the bone marrow. The relatively high signal in the liver observed with radiometal-labeled antibodies reflects progressive antibody degradation and intracellular accumulation of the radiometal (32).

## DISCUSSION

Immuno-PET with bispecific antibodies offers great diagnostic potential alongside emerging AD immunotherapies, enabling more accurate imaging of the targeted Aβ pathology (33), as well as important insights into the brain distribution of next-generation bispecific antibody therapies, such as trontinemab. However, the dual targeting of Aβ and TfR by bispecific antibodies adds complexity to the design and interpretation of brain distribution and PET imaging analyses, and the outcomes are significantly influenced by the choice of radionuclide. Here, we compared Lec-Fab8D3 labeled with ^89^Zr, ^64^Cu, and ^124^I in a mouse model of Aβ pathology to assess how radionuclide selection affects imaging performance.

For all 3 radionuclides, PET imaging with Lec-Fab8D3 showed that the latest time points (48–72 h after radiotracer administration) resulted in the highest distinction in brain uptake and brain-to-blood ratio between Tg-ArcSwe and WT mice because of the slow blood clearance of antibodies. PET images and quantification showed higher overall brain concentrations of Lec-Fab8D3 radiolabeled with the metals ^89^Zr and ^64^Cu, compared with its radioiodinated version in both Tg-ArcSwe and WT mice, whereas all antibody variants displayed a similar absolute difference between the mouse genotypes. This phenomenon has been observed in previous studies of brain-targeted antibodies labeled with radiometals (9,32,34,35). The high retention in WT mice can likely be explained by TfR-mediated internalization of the bispecific antibody into cells such as brain endothelial cells or neurons, which express TfR on their surfaces. After internalization, the antibodies will be degraded. However, because of the residualizing properties of radiometals, the radiotracer will accumulate in the cell rather than being secreted, which leads to a gradually increased nontarget specific retention. Iodine, on the other hand, does not residualize and will therefore be excreted rapidly, which reduces the overall retention time (32,35). Ex vivo γ-counting of isolated brains after perfusion and autoradiography images strengthen this hypothesis further by revealing a higher retention in WT mice who received radiometal-labeled antibody compared with WT mice injected with radioiodinated Lec-Fab8D3. This difference is particularly striking in the cortical region, a highly vascularized area where neurons have a high metabolic activity (36). The residualizing effect was also obvious in peripheral organs, where an approximately 10-fold higher overall radioactive signal was recorded in mice injected with radiometal-labeled compared with radioiodinated Lec-Fab8D3. Interestingly, whereas [^89^Zr]Zr-DFO*-Lec-Fab8D3 remained stable in Tg-ArcSwe mouse brain over time, [^64^Cu]Cu-NODAGA-Lec-Fab8D3 showed a subtle decline between 24 and 48 h. This may be because of only moderate residualizing properties of [^64^Cu]Cu-NODAGA-Lec-Fab8D3, in contrast to the strongly residualizing nature expected for [^89^Zr]Zr-DFO-Lec-Fab8D3 (37,38). Additionally, mice injected with [^64^Cu]Cu-NODAGA-Lec-Fab8D3 showed greater variability, which may be partly attributable to the low amount of radiation remaining after 4 half-lives of decay, challenging the detection accuracy.

Radiometal accumulation in regions that lack the target increases the background activity, which reduces contrast and impairs detection of low-abundance targets across brain regions. This effect is also evident when comparing brain-to-cerebellum ratios in this study, with the cerebellum being used as the reference region because of its lower Aβ burden (7,38). Whereas [^124^I]I-Lec-Fab8D3 showed significantly higher PET-derived brain-to-cerebellum ratios in Tg-ArcSwe mice than in WT mice, both radiometal-labeled antibodies yielded ratios near unity, with no genotype-dependent differences. Ex vivo brain-to-cerebellum ratios and autoradiography, both of which exclude the blood signal, revealed higher cortical versus cerebellar concentrations in Tg-ArcSwe mice injected with [^89^Zr]Zr-Lec-Fab8D3, apparently reflecting Aβ accumulation and leading to a higher brain-to-cerebellum ratio. However, WT mice exhibited an ex vivo brain-to-cerebellum ratio comparable to that of Tg-ArcSwe mice, again associated with an elevated signal in the cortex, but likely from antibody accumulation in cells rather than Aβ binding.

Regional analyses of PET data showed less distinction between genotypes for the brainstem than for the cerebellum, which prompted the inclusion of region-of-interest–to-brainstem analyses alongside the cerebellar normalization. This resulted in greater differences between genotypes for all radiotracers, supporting the suitability of the brainstem as a reference region. However, clear and statistically significant genotype differences in regional ratios were observed only for [^124^I]I-Lec-Fab8D3, reinforcing the strong ability of this radiotracer to discriminate regional differences in Aβ pathology.

Although ^89^Zr- and ^64^Cu-labeled tracers clearly distinguished Tg-ArcSwe from WT and generated higher overall signals than radioiodine, they appear more sensitive to the balance between the dual targets of the bispecific antibody. The simultaneous presence of Aβ and TfR in the brain causes competition for antibody binding, which will then depend on the relative abundance of each target. Since Lec-Fab8D3 exhibits 10- to 100-fold higher affinity for Aβ, the signals in Aβ-rich areas likely reflect true target binding. In contrast, substantial intracellular radiometal accumulation may occur in TfR-rich regions with low Aβ concentrations. Importantly, many brain regions express both targets to varying degrees, which complicates the interpretation of their respective contributions.

All 3 investigated radionuclides—^89^Zr, ^64^Cu, and ^124^I—have been used in immuno-PET for imaging targets within the brain but have not been compared systematically (6–9,39–41). This study shows that Lec-Fab8D3 labeled with ^89^Zr, ^64^Cu, or ^124^I allows imaging of Aβ in the AD mouse brain but that the choice of radionuclide has to be carefully considered. Both ^89^Zr and ^124^I appeared to be highly suitable for immuno-PET, making the choice of radionuclide mainly dependent on factors such as availability, dosimetry, and desired imaging properties. Although ^124^I gave a cleaner picture of Aβ pathology and allowed regional analyses, ^89^Zr provided a higher absolute signal and information about the accumulated off-target antibody distribution in the brain, which may have important implications for therapeutic development. In addition, radiometals could be the preferred radionuclide for brain targets that internalize upon binding, such as glial receptors (39).

## CONCLUSION

PET imaging with Lec-Fab8D3 allowed for sensitive detection of Aβ pathology with radionuclide-dependent imaging outcomes, where ^89^Zr gave the highest absolute signal and ^124^I the highest regional contrast. Our findings underscore the emerging role of immuno-PET with bispecific antibodies as a valuable tool in the development of companion diagnostics for anti-Aβ immunotherapies and further emphasize the importance of radionuclide selection for brain immuno-PET.

## DISCLOSURE

This study was funded by the Swedish Research Council (2021-01083, 2021-03524, and 2024-02963), Alzheimerfonden, Hjärnfonden, Åhlén-stiftelsen, Magnus Bergvalls stiftelse, Konung Gustaf V:s och Drottning Victorias frimurarestiftelse, Tore Nilsons Stiftelse, Stohnes stiftelse, and Stiftelsen för Gamla tjänarinnor. No other potential conflict of interest relevant to this article was reported.

## Notes

KEY POINTS **QUESTION:** Will radionuclide choice influence imaging outcomes in brain immuno-PET with bispecific antibodies? **PERTINENT FINDINGS:** The bispecific, brain-penetrating Aβ antibody Lec-Fab8D3 was labeled with ^89^Zr, ^64^Cu, or ^124^I to systematically compare immuno-PET performance for imaging Aβ pathology. Because of its residualizing properties, ^89^Zr was associated with highest overall brain uptake but produced a prominent background signal in brain tissue devoid of Aβ, whereas the nonresidualizing ^124^I yielded a lower background signal and a greater contrast between brain regions with high versus low Aβ deposition. **IMPLICATIONS FOR PATIENT CARE:** Successful clinical translation of brain-targeted immuno-PET will require careful radionuclide selection, considering the specific properties of both the target and the tracer to optimize imaging performance and diagnostic accuracy.
